# Expanding the phenotype associated to *KMT2A* variants: overlapping clinical signs between Wiedemann–Steiner and Rubinstein–Taybi syndromes

**DOI:** 10.1038/s41431-020-0679-8

**Published:** 2020-07-08

**Authors:** Elisabetta Di Fede, Valentina Massa, Bartolomeo Augello, Gabriella Squeo, Emanuela Scarano, Anna Maria Perri, Rita Fischetto, Francesco Andrea Causio, Giuseppe Zampino, Maria Piccione, Elena Curridori, Tommaso Mazza, Stefano Castellana, Lidia Larizza, Filippo Ghelma, Elisa Adele Colombo, Maria Chiara Gandini, Marco Castori, Giuseppe Merla, Donatella Milani, Cristina Gervasini

**Affiliations:** 1 Genetica Medica e Biologia Applicata, Dipartimento di Scienze della Salute, Università degli Studi di Milano, Milano, Italy; 2 “Aldo Ravelli” Center for Neurotechnology and Experimental Brain Therapeutics, Università degli Studi di Milano, Milano, Italy; 3 Unità di Genetica Medica, IRCSS Casa Sollievo della Sofferenza, San Giovanni Rotondo, Italy; 4 Ambulatorio di Malattie Rare, Sindromologia ed Auxologia U.O. Pediatria AOU S.Orsola-Malpighi, Bologna, Italy; 5 U.O.C. Malattie Metaboliche Genetica Medica, PO Giovanni XXIII, AOU Policlinico Consorziale, Bari, Italy; 6 Centro Malattie Rare e Difetti Congeniti, Fondazione Policlinico Universitario A. Gemelli, Università Cattolica, Roma, Italy; 7 Dipartimento di scienze per la promozione della salute e la cura della madre e del bambino “G. D’Alessandro”, Università di Palermo, Palermo, Italy; 8 Dipartimento di clinica pediatrica e malattie rare, Ospedale pediatrico Antonio Cao, Cagliari, Italy; 9 Unit of Bioinformatics IRCSS Casa Sollievo della Sofferenza, San Giovanni Rotondo, Italy; 10 Research Laboratory of Medical Cytogenetics and Molecular Genetics, IRCCS Istituto Auxologico Italiano, Milan, Italy; 11 Dipartimento di Scienze della Salute, Università degli Studi di Milano, Milano, Italy; 12 UOSD Pediatria ad alta intensità di cura, Fondazione IRCCS Cà Granda Ospedale Maggiore Policlinico Milano, Milano, Italy

**Keywords:** Epigenetics, Mutation

## Abstract

Lysine-specific methyltransferase 2A (KMT2A) is responsible for methylation of histone H3 (K4H3me) and contributes to chromatin remodeling, acting as “writer” of the epigenetic machinery. Mutations in *KMT2A* were first reported in Wiedemann–Steiner syndrome (WDSTS). More recently, *KMT2A* variants have been described in probands with a specific clinical diagnosis comprised in the so-called chromatinopathies. Such conditions, including WDSTS, are a group of overlapping disorders caused by mutations in genes coding for the epigenetic machinery. Among them, Rubinstein–Taybi syndrome (RSTS) is mainly caused by heterozygous pathogenic variants in *CREBBP* or *EP300*. In this work, we used next generation sequencing (either by custom-made panel or by whole exome) to identify alternative causative genes in individuals with a RSTS-like phenotype negative to *CREBBP* and *EP300* mutational screening. In six patients we identified different novel unreported variants in *KMT2A* gene. The identified variants are *de novo* in at least four out of six tested individuals and all of them display some typical RSTS phenotypic features but also WDSTS specific signs. This study reinforces the concept that germline variants affecting the epigenetic machinery lead to a shared molecular effect (alteration of the chromatin state) determining superimposable clinical conditions.

## Introduction

Lysine-specific methyltransferase 2A (*KMT2A*, OMIM +159555) located at 11q23.3 contributes to chromatin opening, acting as “writer” of the epigenetic machinery. This gene encodes for a DNA-binding protein that methylates lysine 4 of histone H3 (K4H3me), which positively regulates transcription of multiple genes, including those involved in hematopoiesis and neuronal development [1]. *KMT2A* is expressed ubiquitously in human tissues and has been broadly studied in hematological diseases, since somatic translocations at its locus characterize a subset of acute leukemias [2]. More recently, heterozygous *KMT2A* loss-of-function and missense variants have been associated to Wiedemann–Steiner syndrome (WDSTS, OMIM #605130), described for the first time in 1989 by Wiedemann et al. [3] and characterized by hypertrichosis *cubiti*, intellectual disability (ID) and developmental delay, a distinctive facial appearance and short stature [4].

Since 2012, pathogenic variants have been reported in patients with a typical WDSTS phenotype [4–11] and most of them generate a truncated protein. Additional variants have been annotated by exome sequencing performed for undiagnosed genetic disorders (https://decipher.sanger.ac.uk/). Studies in large cohorts of syndromic/non-syndromic ID and neurodevelopmental disorders with heterogeneous phenotype have led to the identification of further 32 *KMT2A* novel variants [12–15]. In addition, Li et al. have described 11 unreported mutations in a study comparing French and Chinese cohorts [15, 16]. Besides WDSTS, deleterious variants in *KMT2A* were also described in association with epilepsy [17], primary immunodeficiency [18], and eosinophilia [19].

Interestingly, five more patients carrying *KMT2A* variants were clinically diagnosed with Coffin–Siris syndrome (CSS, OMIM #135900, #614608, #614607, #614609, #616938, #615866, #617808, #618362, #618027, #618506) (pt #K2431) [20], Cornelia de Lange syndrome (CdLS, OMIM #122470, #300590, #610759, #614701, #300882) (pt CdLs #3 and pt #1) [21, 22], Kabuki syndrome (KS, OMIM #147920, #300867) (pts #KS8-KS29) [23], and Rubinstein–Taybi syndrome (RSTS, OMIM #180849, #613684) (pt #103) [24] respectively (Supplementary Table S1). To note, all the above-mentioned syndromes and WDSTS are typical epigenetic machinery disorders, also called chromatinopathies, a group of diseases caused by alterations in genes coding for components of the epigenetic apparatus termed “writers, erasers, readers, and remodelers” [25]. These proteins act in concert to control the chromatin opening and closing thus regulating gene expression by modification (i.e., methylation, acetylation, etc.) of histones and DNA.

As well as KMT2A, protein products of RSTS causative genes, CBP and p300, are “writers” of the epigenetic machinery. Both are ubiquitously expressed homologous proteins belonging to the KAT3 family of lysine acetyltransferases [26]. CBP and p300 are encoded by CREB binding protein (*CREBBP*, also known as “CBP”) and E1A-associated protein p300 (*EP300*) respectively. Mutations in the “major gene” *CREBBP*, located at 16p13.3, are responsible for ~60% of RSTS patients [27], while almost 10% of RSTS cases have been diagnosed for pathogenic variants in *EP300*, mapping on chromosome 22q13.2 and called the “minor gene” [28]. RSTS is a rare (1:125,000) autosomal-dominant disorder which, generally, occurs *de novo* [29] and displays phenotypically heterogeneous variability. RSTS peculiar clinical picture includes postnatal growth deficiency, microcephaly, specific dysmorphisms, and skeletal abnormalities (e.g., broad thumbs and big toes). All these signs are hallmarks frequently used for clinical diagnosis, with a wide spectrum of multiple congenital anomalies [30].

To investigate the molecular basis of patients with a suspicious of RSTS diagnosis but without causative variants in *CREBBP* and *EP300*, we performed a whole exome/targeted next generation sequencing (NGS) identifying six different novel variants in *KMT2A*. The identified variants are *de novo* in at least four out of six tested patients (parents are unavailable in two families) and unreported. The clinical reevaluation of patients shows that they display some typical RSTS features but also various WDSTS specific signs with a peculiar global clinical presentation suggesting that different alterations of epigenetic machinery genes can converge to the same molecular effect (alteration opening/closing chromatin) to determine overlapping clinical conditions.

## Materials and methods

### Subjects

Individuals with RSTS initial diagnosis were clinically assessed and diagnosed by the respective clinical geneticist to enter genetic analysis and then carefully phenotypically reevaluated by a single geneticist with a deep expertise in RSTS diagnosis (DM). Informed consent was obtained from all enrolled subjects and families and this study was approved by the local institution ethical committee and review board.

### *CREBBP* and *EP300* molecular analysis

A molecular multi-method approach based on a combination of multiple ligation-dependent probe amplification (MLPA) to detect whole gene or intragenic deletions and direct Sanger sequencing to identify point variants was applied. Genomic DNA was extracted from peripheral blood samples in EDTA using DNeasy Blood and Tissue Kit (Sigma-Aldrich, St. Louis, MO, USA) for MLPA analysis, or with Wizard^®^ Genomic DNA Purification Kit (Promega, Madison, WI, USA) for sequencing. MLPA analysis was performed with the SALSA MLPA commercially available kits (P313-A for *CREBBP* and P333-A1 for *EP300*) (MRC-Holland, Amsterdam, NL) according to the manufacturer’s protocol. Amplified products were separated by capillary electrophoresis using an ABI PRISM 3700 Genetic Analyzer (Applied Biosystems, Foster City, CA, USA) and data were visually inspected using GeneMaker software v.1.5 (SoftGenetics, LLC, State College, PA, USA) and then deeply analyzed using Coffalyser (MRC-Holland). *CREBBP* amplicons encompassing coding regions and splicing junctions were amplified from the genomic DNA of patients #208 and #221 using GoTaq Flexi DNA Polymerase (Promega) and primer’s couples were designed with the public software Primer3 (http://bioinfo.ut.ee/primer3-0.4.0/) and OligoAnalyser 3.1 (https://eu.idtdna.com/calc/analyzer). Amplified fragments were sequenced with the same primers using the Big Dye Terminator v.1.1 or v.3.1 Cycle Sequencing Kit (Applied Biosystems) and the ABI PRISM 3130 sequencer (Applied Biosystems) according to the manufacture’s protocol. Electropherograms were analyzed with ChromasPro software 1.7.5 (Technelysium Pty Ltd., Queensland, AUS) considering the NG_009873.2 sequence as reference for *CREBBP*.

### Multigene panel sequencing and data analysis

All the patients here described, except patient #187, have been tested by multigene panel sequencing. A customized HaloPlex Target Enrichment NGS panel (Agilent Technologies, Santa Clara, CA, USA) was generated using Agilent’s SureDesign tool available at www.agilent.com/genomics/suredesign. A library of all the coding exons and intron–exon boundaries of 68 known genes associated with more than 40 OMIM entries, selected as classified as known chromatinopathies [25], was prepared using the HaloPlex HS Target Enrichment System (Agilent Technologies). Total genome target spanned 384 kb and was theoretically estimated to be completely covered (99.82%). A total of 17,470 amplicons were generated. Libraries were prepared according to the manufacturer’s protocol using the human reference genome GRCh37/hg19. The library was then amplified by PCR to produce a sequencing-ready, target-enriched sample. Fragmented gDNA and final libraries were evaluated using High Sensitivity D1000 ScreenTape System on TapeStation 2200 (Agilent Technologies) and Qubit dsDNA Hs Assay Kits on Qubit^®^ 4 Fluorometer (Thermo Fisher Scientific, Waltham, MA, USA) to quantify, according to the manufacturer’s instructions. Sequencing was performed on Illumina MiSeq System, using the MiSeq Reagent Kit V2-500 cycles (Illumina, San Diego, CA, USA), yielding 151 bp-long paired-end reads and achieving an average read depth of at least 400× for 11–22 samples and at least 20× per-base coverage in more than 98% of the targeted regions. Quality of sequences was preliminary checked with FastQC and trimmed using Trimmomatic (PMID: 24695404) if the quality of at least half-read was lower than 10 (phred quality score). Residual adapter sequences were removed by cutadapt (https://cutadapt.readthedocs.io/en/stable/). Preprocessed reads were aligned against the GRCh37/hg19 reference genome sequence by BWA-0.7.17 software. Depth of coverage statistics for the target regions were calculated by means of TEQC (10.18129/B9.bioc.TEQC) and other custom scripts. Variants were identified by means of the Haplotype Caller tool of GATK v. 3.8 and were annotated with ANNOVAR, using RefSeq gene and transcript annotations (updated to Dec 2016). Variants were sought in the most important public collections, including dbSNP v. 150, ExAC v. 0.3, Exome Variant Server, gnomAD, HRC, Kaviar and ClinVar. Sequencing artefacts were controlled by matching individual-specific variants against an internal database of variants. Missense variants were fully annotated using dbNSFP v3.5 from which we have retrieved precomputed pathogenicity predictions and evolutionary conservation predictions and measures. The significance of candidate variants was classified according to American College of Medical Genetics and Genomics (ACMG) criteria using InterVar (http://wintervar.wglab.org/) (10.1016/j.ajhg.2017.01.004) and Varsome (https://varsome.com/) (10.1093/bioinformatics/bty897) tools.

### Exome sequencing

Patient #187 and his parents has been tested by exome sequencing. Genomic DNA was extracted from whole blood for DNA library preparation and exome enrichment using the Agilent SureSelect V7 kit according to manufacturer instructions. Quality of post-amplification libraries was assessed using DNA 1000 chips on the BioAnalyzer 2100 (Agilent) and Qubit fluorimetric quantitation using Qubit dsDNA BR Assay Kits (Invitrogen by Thermo Fisher Scientific). An indexed 150 bp paired-end sequencing run was performed on an Illumina HiSeq 3000 instrument at CRS4 NGS facility. This approach achieved a 75× average coverage over the 36 Mb of genomic regions sequenced, with 97% regions covered at least 10×. Data analysis has been performed using an analysis pipeline based on public tools. In brief, paired-end sequence reads were aligned to the human genome (hg19) with the Burrows–Wheeler Aligner (BWA MEM) using default settings. Initial mappings were processed using the GATK framework (v4.1.0.0) according to their Best Practices recommendations. Variant sites were identified using GATK Haplotype Caller module. Variants were classified as known or novel based on dbSNP146 and annotated using KGGSeq. Annotations were retrieved from dbNSFP v.3.0 and included positions in UCSC, RefGene, GENCODE, and ENSEMBL transcripts, OMIM and ClinVar annotations, allele frequency in dbSNP, ESP6500 (release SI-V2), 1000 Genomes Project (release 05/2013), ExAC and gnomAD, functional predictions for the amino-acid changes according to different models (SIFT, PolyPhen2, LRT, Pathogenic variantTaster, Pathogenic variantAssessor, and FATHMM). We filtered the identified variants according to recessive/dominant/*de novo* pattern of inheritance, gene features and MAF < 1% using as references dbSNP138, dbSNP141, 1000 Genomes, ESP6500, ExAC, and gnomAD. Subsequently, variants were evaluated for their phenotypic and biological impact.

### Variants validation and segregation analysis

Variants of interest were confirmed by Sanger sequencing following PCR amplification and segregation analysis was performed in the available family trio. PCR reactions were ruled following the protocol for GoTaq Flexi DNA Polymerase (Promega) with patients’ and variants’ specific primer sets considering NG_027813.1 as reference for *KMT2A* gene. The same pairs of primers were used for Sanger sequencing. *KMT2A* exons are numbered like in ENST00000534358.8 reference. Sequence variants were described according to HGVS nomenclature guidelines (https://varnomen.hgvs.org/) and reported in LOVD (https://databases.lovd.nl/shared/genes/KMT2A) as individual IDs #00275545 (pt #208), #00275547 (pt #250), #00275548 (pt #187), #00275549 (pt #251), #00275550 (pt #243), and #00275551 (pt #221).

## Results

### *KMT2A* detected variants

*CREBBP* and *EP300* MLPA analysis was previously performed for all the six described patients and resulted negative. Patients #187, #208, and #221 underwent also *CREBBP* direct sequencing, without detecting pathogenic variants. Hence, we performed NGS analysis with a custom-made panel comprising 68 genes known as causative of chromatinopathies [25], including *CREBBP* and *EP300* genes, to 30 patients, including all the patients here described, except patient #187. NGS exome analysis was applied to #187 trio and additional nine patients. We detected in six patients (five out of 30 tested by multigene panel sequencing and one out of 10 analysed by WES) a novel heterozygous variant in *KMT2A* (Table 1).

**Table 1:** *KMT2A* pathogenic variants and clinical signs of six described patients compared with typical features of RSTS and WDSTS. Sign + means present, sign − absent and sign +/− present in a few cases.

	RSTS	WDSTS	RSTS-208	RSTS-250	RSTS-187	RSTS-251	RSTS-243	RSTS-221
*KMT2A* pathogenic variant NG_027813.1 (NM_001197104.2) GRCh37/hg19			c.553C>T p.(Arg185*)	c.1697_1717dup p.(Leu566_Leu572dup)	c.3596G>A p.(Trp1199*)	c.3603del p.(Ser1202Profs*12)	c.3632_3634+2del p.?	c.3897_3900dup p.(Leu1303Serfs24*)
Position			chr11:118342427	chr11:118343566	chr11:118350915	chr11:118350921	chr11:118350948	chr11:118352691
Exon			3	3	6	6	6	7
Inheritance			*De novo*	NA (unavailable parents)	*De novo*	*De novo*	*De novo*	NA (adopted child)
Date of birth			22/10/2006	17/09/2003	01/01/2002	16/07/1994	20/07/1993	12/07/2010
Sex			M	M	M	F	M	M
Dysmorphisms								
Long eyelashes	89% +	81% +	+	+	+	+	+	+
Synophrys	5–30%+/−	>30% +	−	−	+	−	+	+
Ptosis	<82% +	31% +	+	+	−	+	+	−
Downslanting palpebral fissures	75% +	64% +	+	+	−	–	+	+
Thick eyebrow	>30% +	64% +	+	−	+	+/−	+	–
Narrow palpebral fissures	−	74% +	+	+	−	+	+	+
Hyperthelorism	5% +/−	71% +	+	−	Telecanthus	+	Telecanthus	+
Columella below the alae nasi	89% +	−	+	+	+/−	+	NA	+
Wide nasal bridge	−	68% +	+	+	−	+	+	−
Grimacing smile	80% +	−	+	−	+/−	−	NA	+
High-arched palate	75% +	>30% +	+	NA	NA	−	+	+
Micrognathia	57% +	>30% +	−	+	−	−	+	+
Low set ears	>30% +	46% +	+	+	+/−	−	−	–
Strabismus	60–71%+	−	−	−	−	−	+	−
Flammeus nevus/angioma	5–30%+/−	−	−	NA	−	−	−	NA
Growth failure								
IUGR	−	>30% +	−	−	−	NA	−	Unknown
PNGR	73% +	38% +	+	−	+/−	NA	+	+
Intellectual disability	98% +	98% +	+++	++	+	+	+	+++
Speech delay/absence	90% +	82% +	−	+	+	+	+	+
Behavioral problems	41% +	42% +	+	+	+		++	NA
Vision problems								
Myopia	9% +/−	−	−	−	−	NA	−	−
Teeth anomalies	>30% +	−	−	−	NA	NA	+	−
Musculoskeletal anomalies								
Broad thumbs	91% +	−	−	+	+/−	−	+	+
Angulated thumbs	42% +	−	+	−	−	−	−	+
Broad halluces	92% +	−	+	+	+	−	+	+
Clinodactyly	5–30%+/−	23% +/−	−	−	−	−	+	−
Brachydactyly	5–30%+/−	45% +	−	−	−	−	−	+
Microcephaly	63% +	−	−	+ (Relative)	−	NA	−	+
Delayed bone age	74% +	34% +	−	NA	−	NA	−	−
Hypotonia	70% +	73% +	−	NA	−	NA	+	+
Organ anomalies								
Cryptorchidism	78–100%+	−	−	−	−	−	−	Mobile testis
Heart defect	24–38%+/−	29% +/−	−	−	−		−	−
Brain anomalies								
Abnormal corpus callosum	17% +/−	14% +/−	−	NA	−	NA	−	−
Seizures	25% +/−	9% +/−	−	NA	−	–	−	−
Hirsutism	>30% +	>30% +	−	–	+	NA	+	+
Keloids/naevi	24% +/−	−	−	NA	−	−	−	−
Pilomatricoma	5–30%+/−	−	−	NA	–	–	–	−
Frequent infections	75% +	−	−	NA	–	NA	−	−
Feeding problems	80% +	53% +	−	NA	−	NA	+	+
Gastroesophageal reflux	68% +	−	−	NA	+	NA	−	−
Others			Prominent eyes; thin upper lip; C2–C3 vertebral fusion; cerebellar vermis hypoplasia; organomegaly	Thin upper lip; autism spectrum disorder; epileptiform abnormalities in the left fronto-temporal region	Early puberty (treated)	Thin lips	Thin lips; hearing loss; altered fine motor abilities; born at 35 weeks; pregnancy with a threatened miscarriage	Thin lips; kyphosis; abnormal ears

*NA* not assessed.

+ = Present (>30%).

− = Absent (unreported).

+/− = Present in a few cases (5–30%).

In particular, patient #208 was found carrier of a *de novo* nucleotide substitution in exon 3: c.553C>T, predicted to cause a premature stop codon p.(Arg185*); patient #250 shows a 21 nucleotides insertion in exon 3: c.1697_1717dup, resulting in an aminoacidic in frame duplication p.(Leu566_Leu572dup). Patient #250 parents’ DNA is not available for further testing.

Patients #187, #251, and #243 are carriers of three different *de novo* variants in exon 6: a single nucleotide change c.3596G>A leading to a premature stop codon p.(Trp1199*) in patient #187, a single nucleotide deletion c.3603del, causing a frameshift p.(Ser1202Profs*12) in patient #251 and a five nucleotides deletion c.3632_3634+2del involving the last three nucleotide of exon 6 and the first two bases of the following intronic sequence, suggesting a possible effect on the correct splicing, in patient #243.

Finally, patient #221 is carrier of a four nucleotides duplication in exon 7 c.3897_3900dup, leading to a frameshift p.(Leu1303Serfs24*). The patient is adopted, making impossible verifying the *de novo* origin of this variant.

According to ACMG guidelines, the clinical significance of the variants is pathogenic for patients #208, #187, #251, and #243 as all the variants meet the same ACMG criteria (PVS1, PM2, and PP3). Variant detected in patient #250 has been classified with uncertain significance (PM2, PM4, and BP4), probably due to the lack of predicted impact on gene product, while patient #221 was found carrier of a likely pathogenic variant (PVS1, PM2).

### Clinical phenotypes

#### Patient #208

The patient is a 13 years old boy, born with normal birth parameters. Postnatal growth deficiency was reported. Minor facial anomalies were evident such as downslanting palpebral fissures, columella below the alae nasi, grimacing smile and hypertelorism, broad nasal bridge, long eyelashes, and prominent eyes. Angulated thumbs and broad halluces are present. He shows severe ID and behavioral issues, with aggression. In addition, C2–C3 vertebral fusion, organomegaly, and cerebellar vermis hypoplasia were reported.

#### Patient #250

Patient 250 is a 16 years old boy. A RSTS diagnosis was suspected for the presence of dysmorphisms such as columella below the alae nasi, grimacing smile, and for the skeletal anomalies of hand and feet (broad thumbs and halluces). Unfortunately, parents of patient 250 refuse a clinical reevaluation of their son and are unavailable to give their DNA making impossible test the *de novo* origin of *KMT2A* variant, detected in the son.

#### Patient #187

Patient 187 is a 17 years old boy, born after an uneventful pregnancy with normal birth parameters (weight 3.045 kg, length 47 cm; occipitofrontal circumference (OFC) 33.5 cm, APGAR 8/9). Neonatal hypotonia, a sacral dimple (S4–S5) and postnatal growth deficiency were described. He furthermore had a diagnosis of precocious puberty. Psychomotor development was delayed, with a moderate ID and some behavioral difficulty (anxiety in particular). Cerebral and medullary MRI showed craniocervical junction anomalies with a mild basilar impression. Clinical evaluation pointed out a relative macrocephaly, low anterior hairline, synophrys, long eyelashes, bulbous nose, low set ears, upturned corners of mouth, broad thumbs and halluces, and hirsutism.

#### Patient #251

Patient #251 is a 25 years old girl, born after an uneventful pregnancy with normal birth parameters (weight 3.100 kg). A gastroesophageal reflux was described. Psychomotor development was delayed, with a moderate ID. Cerebral and medullary MRI showed craniocervical junction anomalies with a mild basilar impression. Clinical evaluation pointed out long eyelashes, long and downslanting palpebral fissures, strabismus, epicanthal folds, hypertelorism, high nasal bridge, columella below alae nasi, upturned corners of mouth, broad halluces, and kyphoscoliosis.

#### Patient #243

Patient #243 is a 26 years old boy, born at 35 weeks of gestation. Parents were healthy and non-consanguineous. The pregnancy showed several threatened miscarriages. Weight was 2.750 kg (+0.66 SD), APGAR score 7 and 8 at 1 and 5 min respectively. At the birth: transient polypnea of the newborn and cyanosis, jaundice treated with phototherapy are recorded. Around the age of 6 months, he showed growth and psychomotor retardation and hypotonia. At the auxological evaluation at 10 months of age: length was 67 cm (<3rd centile), weight 6.8 kg, OFC 44.3 cm. GH deficit was diagnosed at 2 years of life and GH therapy was started with good results and continued until 14.5 years. Early puberty was diagnosed at 7 years of life and treated with triptorelin therapy until 9.5 years. Final height was 164.8 cm (3rd centile), target height 171.5 cm (25th centile). At the clinical evaluation hypotonia, hypertricosis, facial dysmorphisms (telecanthus, downslanted and narrow palpebral fissures, and long eyelashes), broad thumbs, and broad distal fingertips were reported. Strabismus has been surgically corrected at 2.5 years of age. Speech language was delayed and he showed learning disability, mild ID and aggressive behavior. At the last examination at 21 years of age aggressive behavior with selective feeding has been confirmed.

#### Patient #221

Patient #221 is a 9 years old male child. He was adopted and the only information on biological family is that the parents were non-consanguineous. He showed dysmorphisms such as coarse face, low anterior airline, synophrys, narrow forehead, hypertelorism, high arched and heavy eyebrows, long eyelashes, narrow and downslanting palpebral fissures, convex nasal bridge, short philtrum, thin lips, high-arched and narrow-palate, micrognathia and abnormal ears, and skeletal anomalies such as brachydactyly, broad and angulated thumbs, broad halluces and kyphosis. Clinical examination also reveals hirsutism, hypertrichosis, mobile testis and anamnestic record disclose feeding problems, postnatal growth retardation, psychomotor delay (hypotonia, delayed erected head, sat and walked independently, and began speaking words at 4 years), and severe ID. At the age of 7.5 years his weight was 22.750 kg (10–25th pc), his height was 106 cm (<−3 SD), and his OFC measured 46.2 cm (<−4 SD). Electroencephalogram and cerebral MRI did not reveal any brain abnormalities; visual evoked potential and auditory brain stem responses to complex sounds (cABRs) were normal too. Ultrasound of the abdomen, ECG, and echocardiography were normal.

Table 1 summarizes the clinical features of six patients here described, comparing the clinical signs of each patient and the typical features of RSTS and WDSTS.

**Fig. 1:**
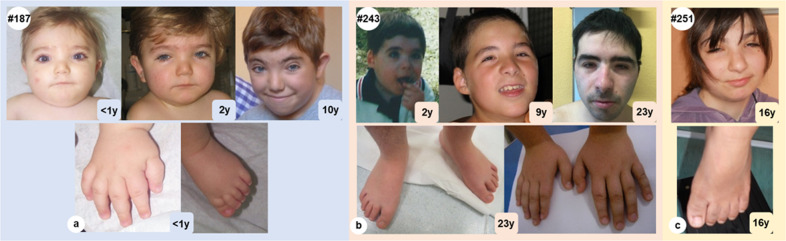
Features of patients #187, #243, and #251, found carriers of *KMT2A* variants. Facies appearance of patient #187 was shown at neonatal age, at 2 and 10 years with particular of hand and foot (**a**). Facies of patient #243 at 2, at 9 and 23 years and hands/feet particulars (**b**). Facies and foot (**c**) particular of patient #251 at 16 years.

There are many clinical signs shared by RSTS and WDSTS patients described in the literature (growth retardation, ID, and specific dysmorphisms). The clinical reevaluation of patients shows that they display some typical RSTS features that appear more evident during childhood, but also various WDSTS specific signs with a peculiar global clinical presentation. All the described patients show the most frequent RSTS signs such as columella below alae nasi (5/5), broad thumbs/hallux (4/6, 5/6), and ptosis (4/6) (Fig. 1), but also typical WDSTS clinical features such as broad nasal tip (4/6), narrow palpebral fissures (5/6), and hirsutism (3/6).

## Discussion

Chromatinopathies define a group of diseases caused by alterations in genes coding for components of the epigenetic apparatus termed “writers, erasers, readers, and remodelers”. These proteins act in concert to control the chromatin opening and closing thus, regulating gene expression by modification (i.e., methylation, acetylation, etc.) of histones and DNA methylation [25].

Due to the clinical overlap between different chromatinopathies and the functional network between the epigenetic components, pathogenic variants in a same “epigene” could be responsible for overlapping and/or slightly different clinical presentation cases.

Accordingly, in a recent work we found that some RSTS-like patients found negative for mutations in the two causative genes turned out to be carriers of variants in other chromatinopathies genes such as *KMT2D* (associated to Kabuki syndrome, KS; OMIM #147920), *ASXL1* (associated to Bohring–Opitz syndrome, BOS; OMIM #605039), and *KMT2A* (associated to Wiedemann–Steiner syndrome WDSTS; OMIM #605130) by WES analysis [24]. These results strongly suggest a possible implication of defective histone-modifying enzymes, other than CBP and p300, in the pathogenesis of RSTS-like phenotype. These findings are in line with the known significant phenotypic overlap observed among Mendelian disorders involving the epigenetic machinery which likely reflects the interconnected pathways involved in the complex regulation of balance between open and closed chromatin [25, 31].

In the present study, we used a custom NGS panel or WES to screen RSTS patients who resulted negative for mutations in *CREBBP* and *EP300* and exome analysis in one trio. We identified six different novel variants in *KMT2A* (5/6 classified as pathogenic/likely pathogenic). Pathogenic variants in this gene, coding for a member of the Lysine methyltransferase 2 family cause the WDSTS.

The identified variants are *de novo* (for the 4/6 patients whose parents are available) and unreported, but indiscernible for type and localization from variants described in WDSTS patients. Interestingly, patients #208 and #221 display a more severe phenotype, although they are carriers of variants predicted pathogenic and likely pathogenic respectively. This is probably due to the involvement of protein domain affected by the *KMT2A* variant, which in patient #208 affects the AT hook domain, while in patient #221 any specific domain is involved. However, lack of a complete molecular picture for patient #221 (as adopted child) unfortunately prevents depth genotype–phenotype correlation. The clinical reevaluation shows that patients display the typical RSTS features, more evident in childhood, but also some WDSTS specific signs with a peculiar global clinical presentation, making difficult the correct diagnosis. WDSTS is a recently defined rare chromatinopathy (first description in 1989) and its clinical signs show several overlapping features with other chromatinopathies. Nevertheless, peculiar clinical signs such as hypertrichosis *cubiti* and a typical facial appearance are present in almost all the WDSTS reported patients [4]. *KMT2A* variants are reported also in one Coffin–Siris (CSS) patient (pt #K2431 in Bramswig et al. [20]), in two Cornelia de Lange syndrome (CdLS) patients (Yuan et al. [21], patient CdLS #3 and Parenti et al. [22], patient #1), in two patients with a diagnosis of Kabuki syndrome (KS) (patients KS8 and KS29 in Sobreira et al. [23]) and in another RSTS-like patient from our previous cohort (Negri et al. [24], pt #103). Recently, several papers [12–15, 17–19] report *KMT2A* pathogenic variants in patients without the “obligate” specific WDSTS clinical feature showing a broader phenotypic spectrum due to *KMT2*A alterations. All the above cited clinical conditions and in particular WDSTS and RSTS show several phenotypic overlapping (e.g., growth deficiency, neurological/cognitive impairment, similar dysmorphisms, and limb anomalies), as observed in our cohort, but we can envisage also a shared molecular cause. In all these cases, in fact, the causative variants affect a gene coding an epigenetic player that, by modulating the interconnected DNA methylation and histone acetylation/methylation, define a correct and site-specific chromatin opening/closing balance. A loss of a specific player could perturb the equilibrium leading to similar consequences or to a spectrum of overlapping clinical conditions.

Reports in literature point out that variants in the same gene can lead to different phenotypes with a set of common features indicative of a group of disorders. On the other side, variants in different genes encoding proteins implicated in the same cellular pathway can lead to the same phenotype. The different clinical outcomes due to variants in the same gene can be attributed not only to specific genotype–phenotype correlations, but also, to stochastic events occurring during development and the different genetic background among patients [20, 32, 33].

In summary, using a NGS-dedicated approach, we found *KMT2A* variants in six patients with an initial RSTS clinical diagnosis, supporting that different mechanisms leading to imbalance of chromatin opening/closing can converge to a similar phenotype characterized by several overlapping RSTS/WDSTS clinical signs. Moreover, these patients show a moderately different spectrum of clinical findings, suggesting that different *KMT2A* variants or allelic heterogeneity could contribute to the wide variability of observed phenotypes of chromatinopathies.

## Supplementary information

Supplementary Table S1
