# Supplementary material for: Expanding the phenotype associated to KMT2A variants: overlapping clinical signs between Wiedemann–Steiner and Rubinstein–Taybi syndromes
Source: Eur J Hum Genet. 2020 Jul 8;29(1):88–98. doi: 10.1038/s41431-020-0679-8 (PMC7852672; doi:10.1038/s41431-020-0679-8)
Supplement: Supplementary file 1 — Supplementary Table S1 [file 41431_2020_679_MOESM1_ESM.docx]

**Supplementary Table S1.** List of variants so far described for *KMT2A* gene.

| **Gene** | **cDNA** | **Protein** | **Diagnosis** | **Reference** |
| --- | --- | --- | --- | --- |
| *KMT2A* | c.74del | p.(Gly26Alafs*2) | WDSTS + eosinophilia | Zhang et al. (2019) |
|  | c.152_186del | p.(Pro51Argfs*84) | WDSTS | Aggarwal et al. (2017) |
|  | c.173dup | p.(Ala59Glyfs*88) | WDSTS | Feldman et al. (2018) |
|  | c.269C>A | p.(Ser90*) | ID | Baer et al. (2018) |
|  | c.(432+1_433-1)_(4332+1_4333-1)del | p.? | WDSTS | Mendelsohn et al. (2014) |
|  | c.478C>T | p.(Arg160*) | ID | Baer et al. (2018) |
|  | c.553C>T | p.(Arg185*) | RSTS-like | This work |
|  | c.654_679delins | p.(Glu219Leufs*27) | ID | Baer et al. (2018) |
|  | c.838C>A | p.(Pro280Thr) | WDSTS | Miyake et al. (2016) |
|  | c.839_843del | p.(Pro280Glnfs*3) | WDSTS | Grangeia et al. (2019) |
|  | c.901C>T | p.(Arg301*) | WDSTS + glossoptosis | Li et al. (2018) |
|  | c.1038del | p.(Val347Leufs*53) | WDSTS | Miyake et al. (2016) |
|  | c.1142dup | p.(Ala383Glyfs*6) | ID | Baer et al. (2018) |
|  | c.1697_1717dup | p.(Leu566_Leu572dup) | RSTS-like | This work |
|  | c.1868del | p.(Lys623Serfs*3) | Undiagnosed genetic condition (WES) | Farwell et al. (2015) reported as p.(L623Sfs*3) |
|  | c.2148del | p.(Leu717Cysfs*39) | WDSTS | Miyake et al. (2016) |
|  | c.2193del | p.(Ser732Profs*24) | Unclassified epilepsy | Helbig et al. (2016) |
|  | c.2233C>T | p.(Arg745*) | CdLS-like | Yuan et al. (2015) |
|  | c.2255dup | p.(His753Serfs*11) | EpiIeptic Encephalopathy; Infant Spasms; Undiagnosed genetic condition (WES) | Helbig et al. (2016); Farwell et al. (2015) |
|  | c.2262del | p.(Met755*) | Epileptic encephalopathy; ESES; complex partial seizures | Helbig et al. (2016) |
|  | c.2318dup | p.(Ser774Valfs*12) | WDSTS + macroencephaly; Undiagnosed genetic condition (WES) | Baer et al. (2018); Farwell et al. (2015); Li et al. (2018) |
|  | c.2455C>T | p.(Gln819*) | WDSTS | Sun et al. (2016) |
|  | c.2510dup | p.(Trp838Ilefs*9) | WDSTS | Li et al. (2018) |
|  | c.2618G>T | p.(Ser873Asn) | ID | Baer et al. (2018) |
|  | c.3019G>T | p.(Gly1007Cys) | KS-like | Sobreira et al. (2017) |
|  | c.3247C>T | p.(Arg1038*) | WDSTS | Dunkerton et al. (2015) |
|  | c.3130_3133del | p.(Gln1045Profs*48) | WDSTS + preaxial polydactyly | Enokizono et al. (2017) |
|  | c.3241C>T | p.(Arg1081*) | WDSTS | Li et al. (2018) |
|  | c.3301C>T | p.(Arg1101*) | ID | Baer et al. (2018) |
|  | c.3334+1G>A | p.? | NDD/ID | Popp et al. (2017) |
|  | c.3460C>T | p.(Arg1154Trp) | ID | Baer et al. (2018) |
|  | c.3464G>A | p.(Cys1155Tyr) | CSS-like; ID | Bramswig et al. (2015); Baer et al. (2018) |
|  | c.3481T>G | p.(Cys1161Gly) | WDSTS | Stellacci et al. (2016) |
|  | c.3503G>A | p.(Gly1168Asp) | WDSTS | Min Ko et al. (2017) reported as c.3504G>A; Li et al. (2018) |
|  | c.3542G>A | p.(Gly1181Asp) | ID | Baer et al. (2018); Li et al. (2018) |
|  | c.3566G>A | p.(Cys1189Tyr) | WDSTS | Miyake et al. (2016) |
|  | c.3581G>A | p.(Cys1194Tyr) | ID | Lebrun et al. (2018) |
|  | c.3596G>A | p.(Trp1199*) | RSTS-like | This work |
|  | c.3603del | p.(Ser1202Profs*12) | RSTS-like | This work |
|  | c.? | p.(Lys1218Glufs*4) | Undiagnosed genetic condition (WES) | Lee et al. (2015) |
|  | c.3632_3634+2del | p.? | RSTS-like | This work |
|  | c.3837del | p.(Pro1281Leufs*75) | WDSTS; Undiagnosed genetic condition (WES) | Li et al. (2018); Hu et al. (2017) |
|  | c.3895_3896del | p.(Ser1299Profs*26) | ID | Baer et al. (2018) |
|  | c.3897_3900dup | p.(Leu1303Serfs*24) | RSTS-like | This work |
|  | c.4012del | p.(Gly1338Valfs*18) | ID | Baer et al. (2018) |
|  | c.4032del | p.(Val1347Thrfs*9) | ID | Baer et al. (2018) |
|  | c.4059del | p.(Pro1354Leufs*2) | WDSTS | Li et al. (2018) |
|  | c.4086+1G>A | p.? | WDSTS | Storm et al. (2014) |
|  | c.4177dup | p.(Ile1393Asnfs*14) | WDSTS | Ramirez-Montano et al. (2019) |
|  | c.4342T>C | p.(Cys1448Arg) | WDSTS | Storm et al. (2014); Lee et al. (2015) |
|  | c.4599dup | p.(Leu1534*) | WDSTS | Jones et al. (2012) |
|  | c.4667_4668del | p.(Cys1556Serfs*2) | ID | Baer et al. (2018) |
|  | c.4696+1G>A | p.? | ID | Baer et al. (2018) |
|  | c.4897C>T | p.(Arg1633*) | WDSTS; ID | Steel et al. (2015); Baer et al. (2018) |
|  | c.5363+1del | p.? | RSTS-like | Negri et al. (2019) |
|  | c.5603del | p.(Pro1868Glnfs*3) | ID | Baer et al. (2018) |
|  | c.5871T>A | p.(Tyr1957*) | WDSTS + dyslipidemia; external ear deformity; carpal epiphyseal growth retardation | Li et al. (2018) |
|  | c.5803-1G>A | p.? | KS-like | Sobreira et al. (2017) |
|  | c.5873A>G | p.(His1958Arg) | ID | Baer et al. (2018) |
|  | c.5932C>T | p.(Gln1978*) | WDSTS | Min Ko et al. (2017) |
|  | c.6002_6005del | p.(Phe2001Trpfs*8) | ID | Baer et al. (2018) |
|  | c.6052del | p.(Glu2018Asnfs*7) | WDSTS | Li et al. (2018) |
|  | c.6079+1G>C | p.? | WDSTS | Feldman et al. (2018) |
|  | c.6080G>A | p.(Gly2027Glu) | ID | Baer et al. (2018) |
|  | c.6379C>T | p.(Arg2127*) | WDSTS + gonadal dysgenesis | Calvel et al. (2015) |
|  | c.6533_6534ins | p.(Val2179Serfs*5) | ID | Martinez et al. (2017) |
|  | c.6487C>T | p.(Arg2163*) | ID | Baer et al. (2018) |
|  | c.6781C>T | p.(Gln2261*) | WDSTS | Miyake et al. (2016) |
|  | c.6913del | p.(Ser2305Leufs*2) | WDSTS | Jones et al. (2012) |
|  | c.7062del | p.(Ser2355Leufs*18) | WDSTS + GHD | Stoyle et al. (2018) |
|  | c.7144C>T | p.(Arg2382*) | WDSTS | Jones et al. (2012) |
|  | c.7264G>T | p.(Gly2422*) | Undiagnosed genetic condition (WES) | Zemojtel et al. (2015) |
|  | c.7438C>T | p.(Arg2480*) | WDSTS; ID | Miyake et al. (2016); Baer et al. (2018) |
|  | c.7630G>T | p.(Glu2544*) | ID | Baer et al. (2018) |
|  | c.7975C>T | p.(Arg2659*) | ID | Baer et al. (2018) |
|  | c.8092del | p.(Glu2698Asnfs*59) | WDSTS | Grangeia et al (2019) |
|  | c.8174_8177del | p.(Asp2725Glyfs*31) | ID | Baer et al. (2018) reported as p.(E2725Vfs*22) |
|  | c.8267del | p.(Leu2756*) | WDSTS | Jones et al. (2012) |
|  | c.8270dup | p.(Ile2758Aspfs*2) | ID | Baer et al. (2018) |
|  | c.8407C>T | p.(Gln2803*) | WDSTS | Sun et al. (2016); Li et al. (2018) |
|  | c.8558T>G | p.(Met2853Arg) | ID | Lebrun et al. (2018) |
|  | c.8590C>T | p.(Gln2864*) | CdLS-like | Parenti et al. (2017) |
|  | c.8806_8809del | p.(Val2936*) | WDSTS | Jones et al. (2012) |
|  | c.9440C>T | p.(Ser3147Phe) | ID | Baer et al. (2018) |
|  | c.9682del | p.(Arg3228Valfs*28) | ID | Martinez et al. (2017) |
|  | c.9714_9735del | p.(Pro3239Leufs*10) | ID | Baer et al. (2018) |
|  | c.9911del | p.(Leu3304Argfs*19) | Undiagnosed genetic condition with short stature (WES) | Homma et al. (2019) |
|  | c.? | p.(Ser3446Phefs*29) | Undiagnosed genetic condition (WES) | Lee et al. (2015) |
|  | c.10343del | p.(Ala3449Glnfs*10) | Undiagnosed genetic condition (WES) | Zemojtel et al. (2015) reported as p.(E3448fs*7) |
|  | c.10752dup | p.(Gly3585Argfs*8) | WDSTS; Undiagnosed genetic condition (WES) | Li et al. (2018); Hu et al. (2017) |
|  | c.10837C>T | p.(Gln3613*) | WDSTS + deep palmar crease | Li et al. (2018) |
|  | c.10835+1G>A | p.? | CVID/WDSTS | Bogaert et al. (2017) |
|  | c.10850T>C | p.(Leu3617Pro) | ID | Baer et al. (2018) |
|  | c.10900+2T>C | p.? | WDSTS | Li et al. (2018) |
|  | c.11322-1G>A | p.? | ID | Lebrun et al. (2018) |
|  | c.11716C>T | p.(Arg3906Cys) | WDSTS | Li et al. (2018) |

CVID= Common variable immunodeficiency

ESES= Electrical status epilepticus during sleep

GHD= Growth hormone deficiency

NDD= Neurodevelopmental disorders
